# Anxiety symptoms and burnout among Chinese medical staff of intensive care unit: the moderating effect of social support

**DOI:** 10.1186/s12888-020-02603-2

**Published:** 2020-05-01

**Authors:** Hui Zhang, ZhiHong Ye, Leiwen Tang, Ping Zou, Chunxue Du, Jing Shao, Xiyi Wang, Dandan Chen, Guojing Qiao, Shao Yu Mu

**Affiliations:** 1 Zhejiang University School of Medicine Sir Run Run Shaw Hospital, Hangzhou, 310016 Zhejiang China; 2 School of Nursing, Nipissing University, 750 Dundas Street West, Toronto, Ontario Canada; 3 Guizhou Provincial People’s Hospital, Guiyang, 550002 Guizhou China; 4 The Affiliated Hospital of Guizhou Medical University, Guiyang, 550001 Guizhou China; 5 Nursing College of Chongqing Medical University, Chongqing, 400016 China

**Keywords:** Anxiety, Social support, Burnout, Intensive care unit, Moderating effects

## Abstract

**Background:**

Social support can be a critical resource to help medical staff cope with stressful events; however, the moderating effect of social support on the relationship between burnout and anxiety symptoms has not yet been explored.

**Methods:**

The final sample was comprised of 514 intensive care unit physicians and nurses in this cross-sectional study. Questionnaires were used to collect data. A moderated model was used to test the effect of social support.

**Results:**

The moderating effect of social support was found to be significant (b = − 0.06, *p* = 0.04, 95%CI [− 0.12, − 0.01]). The Johnson-Neyman technique indicated that when social support scores were above 4.26 among intensive care unit medical staff, burnout was not related to anxiety symptoms.

**Conclusions:**

This is the first study to test the moderating effect of social support on the relationship between burnout and anxiety symptoms among intensive care unit staff.

## Background

Burnout syndrome is defined as “a psychological syndrome emerging as a prolonged response to chronic interpersonal stressors on the job” [1]. The World Health Organization (WHO) classifies burnout as an “occupational phenomenon” in International Classification of Diseases 11th revision (ICD-11) [2]. This syndrome is characterized by three domains: emotional exhaustion, depersonalization, and reduced personal accomplishment. Burnout is attributed to demanding and highly stressful work environments (e.g., longer work hours, insufficient community support in the workplace, and a heavy workload) [3]. Evidence shows that burnout among medical staff is a public health crisis that requires urgent action [4]. Not only can burnout negatively affect the ability of medical professionals to properly care for their patients, such as medical errors, but it may be detrimental to their mental wellbeing (e.g., anxiety and depression) [4]. A systematic review has shown that one of the most important outcomes is psychological problems for employees suffering burnout and burnout is associated with an increased prevalence of anxiety among male and female employees [5].

Due to the elevated levels of workplace tension, physicians and nurses are consistently subjected to mental health issues, such as anxiety, which is defined as “a psychological state typically correlated with uneasiness, fear, or worry” [6]. The economic burden of anxiety is huge and anxiety is considered to be the sixth leading cause of disability [7, 8]. In China, healthcare professionals are placed under great pressure due to poor working conditions, highly stressful relationships between caregivers and patients, and severe medical staff shortages [9, 10]. This also makes Chinese medical staff vulnerable to developing anxiety symptoms. Studies found that the prevalence of anxiety among medical professionals in China was between 25.67–41.1% [10–12]. Poor mental health can have a negative impact on physical health. Evidence shows that mental health problems are responsible for chronic diseases, like cardiovascular disease [13]. Anxiety can also lead to physical symptoms, such as fatigue, sleep problems, and muscle spasms [14]. Medical staff with anxiety have difficulty in concentrating on their clinical practice, resulting in inappropriate medical treatments, thus causing potential gaps in patient care and safety [15].

Social support is defined as “those social interactions or relationships that provide individuals with actual assistance or that embed individuals within a social system believed to provide love, care, or sense of attachment to a valued social group or dyad” [16]. Social support includes both received social support and perceived social support. Received support refers to an individual obtaining actual help from others, whereas perceived support refers to the belief that such helping behaviors will be available when needed [17]. These supports are commonly provided by colleagues, family, and friends [18]. There are many psychological resources that medical professionals can adopt to improve psychological stress and mental health. This study focuses specifically on received and perceived social supports due to their role in protecting against mental health issues [19, 20]. Social support is theorized to impact mental health through two main effects: a direct effect and a buffering effect [18]. The existing empirical literature proposes that social support directly influences the wellbeing of medical staff [21, 22]. Additionally, it is now well supported from a variety of studies that social support has moderating effects. Researchers have found that social support can moderate the effects of stress on mental health [23, 24]. According to Dir et al., (2019) social support has a moderating effect on the relationship between burnout and mental health stigma [25]. The implications of this are that the effects of stressors on mental health can be moderated by the magnitude of social support.

From the empirical evidence, it can be inferred that burnout has a positive relationship with anxiety symptoms. Moreover, social support may moderate the relationship between burnout and anxiety symptoms. However, the moderating effect of social support on the relationship between burnout and anxiety symptoms has not been explored yet. To fill this gap, this study sought to test the moderating effect of social support on the relationship between burnout and anxiety symptoms among staff working in the intensive care unit (ICU). These highly specialized and well-trained professionals work in an especially stressful care setting, where they are constantly exposed to high patient morbidity and mortality, difficult ethical decision-making, and end-of-life issues [26]. These staff are also expected to provide high-quality, multidisciplinary care to critically ill patients despite limited resources [27]. Consequently, critical care staff have one of the highest rates of burnout (> 50%), and are more likely to develop high levels of anxiety or depression [28, 29].

### Theoretical background

According to the Job Demand-Resources Model (JD-R Model), job demands are ‘negative factors’, such as a heavy workload and demanding interactions with clients. In contrast, job resources are ‘positive factors’, such as advanced job skills and enough leisure time [30]. When job demands are chronically high and job resources are limited, burnout can happen [30]. Schaufeli (2017) has proposed an integrative conceptual framework on the basis of the JD-R model [31]. This framework contains two basic processes. The first being the motivational process whereby large amounts of job resources predict work engagement, ultimately leading to positive organizational outcomes, such as organizational commitment. The second process is the stress process, which means that excessive job demands and limited job resources can result in burnout, leading to mental health problems [31]. On the basis of this stress process, anxiety symptoms may be one of the mental health problems occurring in response to burnout. Research has supported the direct effects of burnout on anxiety among Chinese physicians [32]. According to the buffering model of social support, individuals who receive social support are less impaired when they face stressful events [18]. In line with the buffering model of social support, high burnout may be associated with high frequent anxiety symptoms if social support is limited. Therefore, the effect of burnout on anxiety symptoms can be moderated by social support, due to its buffering effects. The moderating effect of social support have been found in the literature [23, 24].

**Fig. 1:**
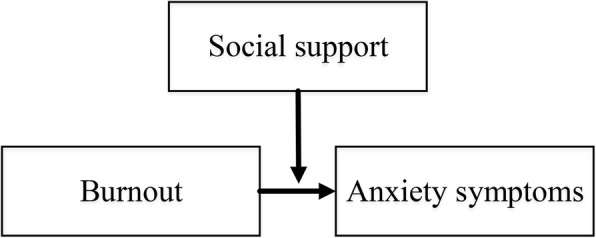
The conceptual model of this research

These theories were integrated to investigate the buffering effect of social support on mitigating the stress process of JD-R Model. This means that social support can moderate the relationships between burnout and anxiety symptoms. Figure 1 shows the conceptual model where burnout is the main predictor, social support is the moderator, and anxiety symptoms are the outcome.

## Methods

### Study units and participants

A convenience sampling method was used to select participants. From November 2018 to March 2019, data collection was done by three trained researchers using anonymous, self-administered questionnaires. ICU nurses and physicians were recruited from Guizhou Provincial People’s Hospital, the affiliated hospital of Guizhou Medical University, and Sir Run Run Shaw Hospital. We contacted all ICU directors and head nurses via email to participate in this study. Once ICU directors or head nurses agreed to the study, they provided the number of physicians and nurses working in their ICU. The researchers explained the aims of the study to ICU medical staff and informed them that the data would be kept confidential. After they made their decisions to participate in this study, the informed consent was obtained. The study was performed in accordance with the ethical principles set forth in the Helsinki Declaration. Each hospital obtained ethics approval from their respective ethics committee.

### Measures

#### Anxiety symptoms

Anxiety symptoms were measured on a 4-point Likert scale with 20 items from the Chinese version of the Zung Self-Rating Anxiety Scale (SAS) [33]. Example items include “I feel afraid for no reason at all”, “I feel more nervous and anxious than usual”, and “I feel like I’m falling apart and going to pieces”. An original total score is multiplied by 1.25 to get the standard score. The present study defined anxiety symptoms as a standard score ≥ 50 [34]. A previous study showed that the reliability of SAS was 0.88 [34].

#### Social support

The Chinese questionnaire of the Social Support Rating Scale (SSRS) was applied to assess social support in this study [35]; it comprises three dimensions: objective support, subjective support, and the usage of support. Example items include “In the past, when you encounter difficulties, what is the source that you ever received either economic support or practical problem-solving help?”, “How many intimate friends do you have, from whom you can receive support and help?”, and “What is the way of seeking help when you are in trouble?”. Participants were asked to score on 4-point Likert scales. The social support scores range from 12 to 66, indicating that higher scores receive more social support. A Chinese study has also suggested SSRS has acceptable internal consistency (Cronbach’s alpha = 0.71) [36].

#### Burnout

The Chinese Burnout Inventory is designed to screen for burnout; it consists of three dimensions: emotional exhaustion, depersonalization, and reduced personal accomplishment. Example items include “I feel emotionally drained from my work”, “I have become more callous toward people since I took this job”, and “I have accomplished many worthwhile things in this job” [37]. Respondents rated their level of agreement with each item on a scale of 0 to 6. The cut-off values for the three dimensions are > 25, 11, and 16, respectively. According to these dimensions, burnout is defined as being at or above the cut-off score in at least one of the three dimensions. A previous study showed that Cronbach’s alpha was 0.816 among Chinese physicians [32].

### Statistical analysis

Pearson correlational analysis and exploratory factor analysis were performed for the three variables (anxiety symptoms, social support, and burnout) using IBM® SPSS® Statistics (Version 24, IBM Corporation, New York, NY). After exploratory factor analysis, the mean values of those variables were used to conduct a PROCESS analysis. To show the prevalence of burnout and anxiety symptoms, these variables were regarded as categorical variables. However, in the PROCESS analysis, social support, burnout, and anxiety symptoms were considered to be continuous variables. PROCESS macro was used in this study, and it was based on ordinary least-squares regression [38]. We adopted the model 1 of PROCESS macro to test the significance of the moderated model. The Johnson–Neyman (J-N) technique was applied for probing interactions [39]. The J-N technique can identify regions in the range of the moderator variable where the effect of burnout on anxiety symptoms is statistically significant or not significant.

## Results

### Preliminary analyses

A total of 615 ICU physicians and nurses from 3 hospitals were invited. Eleven ICU medical staff refused to participate in the study and 604 consented to participate. Five hundred fifty-eight questionnaires were returned, representing a 92.4% response rate. The final sample was comprised of 514 participants, because they completed questionnaires. In total, 56.03% of participants experienced burnout, and 48.25% participants developed anxiety symptoms (Table 1). The descriptive statistics, correlation matrix, Cronbach’s alphas, and average variance extracted (AVE) are shown in Table 2. Acceptable convergent validity was found due to each AVE exceeding 0.50. Because the square root of AVE values for social support (0.74), burnout (0.73), and anxiety (0.85) exceeded the construct correlation values (− 0.26–0.38), the discriminant validity is satisfactory in our study [40].

**Table 1:** Demographic characteristics of study participants (*N* = 514)

	N /Mean	%/(SD)
**Gender**		
Male	132	25.68
Female	382	74.32
**Age (years)**	29.38	5.20
**Work experience (years)**	6.17	5.56
**Marital status**		
Married	300	58.37
Unmarried/Divorce	214	41.63
**Profession**		
Physicians	100	19.46
Nurses	414	80.54
**Burnout**		
No	226	43.97
yes	288	56.03
**Anxiety symptoms**		
No	266	51.75
Yes	248	48.25

**Table 2:** The correlation coefficient, mean, standard deviation, Cronbach’s alphas, and AVE (*N* = 514)

	M	SD	AVE	1	2	3
1.Social support	2.78	0.73	0.56	(0.73)		
2.Burnout	2.98	1.02	0.53	−0.08^a^	(0.81)	
3.Anxiety symptoms	1.75	0.57	0.73	−0.26^a^	0.38^a^	(0.82)

^a^Significant at the 0.01 level; *AVE* average variance extracted; The Cronbach’s alphas are provided in parentheses on the diagonal

### Moderation analyses

**Fig. 2:**
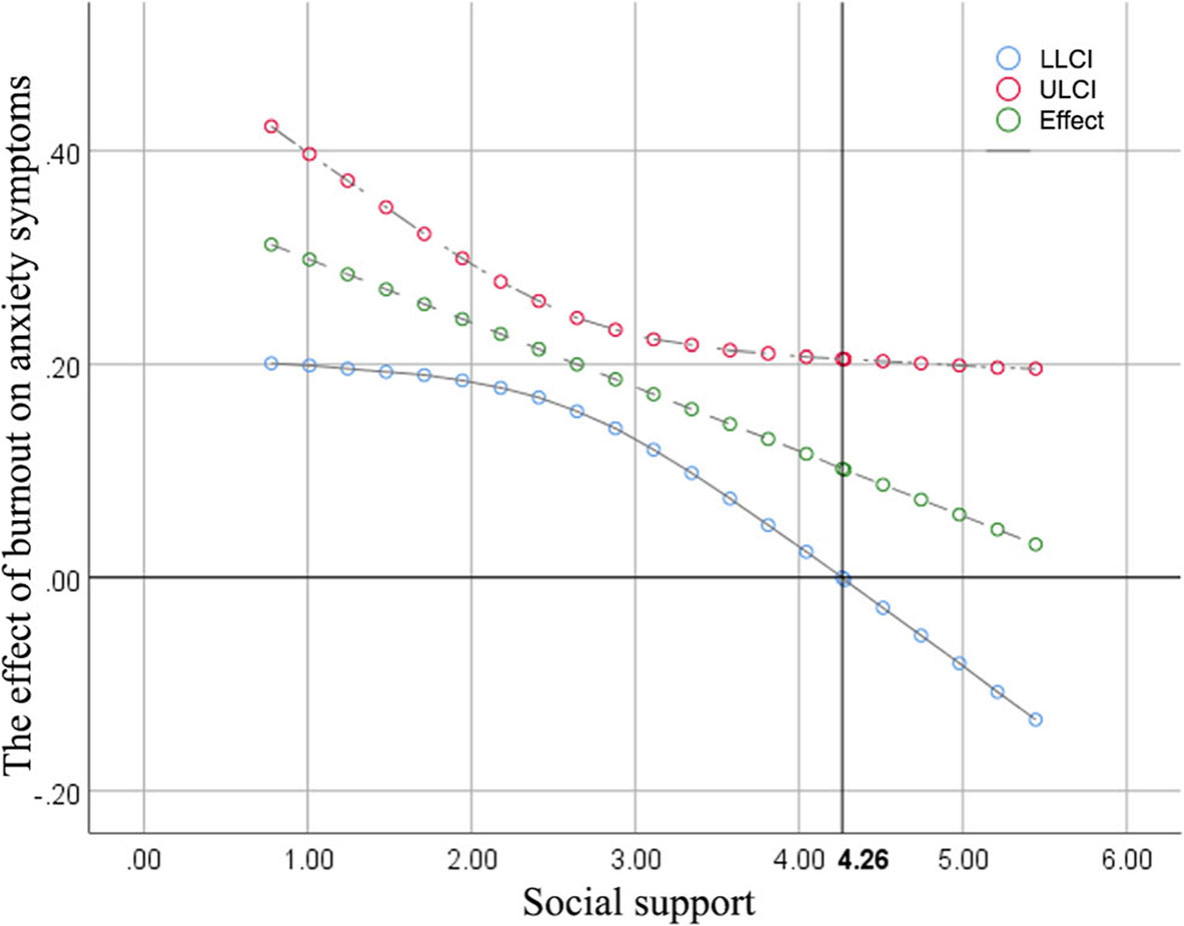
Effect of burnout on anxiety symptoms at different levels of social support. ULCI = Upper Limit of Confidence Interval, LLCI = Lower Limit of Confidence Interval; Effect = the effect of burnout on anxiety symptoms. The horizontal line represents the unstandardized effect of zero, and the vertical line represents the transition value of the moderator

As shown in Table 3, the direct effect of burnout on anxiety symptoms was found to be significant (b = 0.36, *p* < 0.001, 95%CI [0.21, 0.51]), and the moderating effect of social support was also found to be significant (b = − 0.06, *p* = 0.04, 95%CI [− 0.12, − 0.01]). The Johnson-Neyman technique indicated that the score of 4.26 on the social support can be regarded as a point of transition between a statistically significant and a nonsignificant effect of burnout on anxiety symptoms (Fig. 2).

**Table 3:** Moderation analyses

DV	IV	coeff	se	t	*p*	LLCI	ULCI
Anxiety symptoms	constant	1.19	0.25	4.76	*p* < 0.001	0.70	1.68
	Social support	−0.003	0.09	−0.04	0.97	−0.18	0.17
	Burnout	0.36	0.08	4.63	*p* < 0.001	0.21	0.51
	Interaction	−0.06	0.03	−2.11	0.04	−0.12	− 0.01

*DV* Dependent variable, *IV* Independent variable, *ULCI* Upper Limit of Confidence Interval, *LLCI* Lower Limit of Confidence Interval

## Discussion

This study was designed to determine the moderating effect of social support on the relationship between burnout and anxiety symptoms. The results support the hypothesis that social support moderates the relationship between burnout and anxiety symptoms among ICU staff.

A high level of burnout was directly related to high levels of anxiety symptoms as shown in this study. The findings align with the stress process described in the JD-R model outlining burnout can lead to mental health issues. There is a clear link between burnout and psychological strain (e.g., anxiety). In response to burnout, individuals are likely to suffer from anxiety symptoms, which can be regarded as negative outcomes in the stress process [31]. Studies have also found similar results, including Zhou et al. (2018) who found the same relationships between burnout and anxiety symptoms among Chinese nurses [10]. Furthermore, a systematic review by Bakker et al. (2014) confirmed that one of the most important negative consequences of burnout is psychological problems [5]. ICU staff often need to face the life-and-death struggle in a very demanding profession, so they are more likely to be emotionally and physically drained. Under this situation, burnout can pose a threat to psychological problems. It is vital for policy makers to raise awareness about the problems that the directly negative effect of burnout on anxiety symptoms among ICU professionals.

The findings from this study highlight social support can play a key role in mitigating the detrimental effect of burnout on anxiety symptoms among ICU professionals. It has been previously observed that the moderating effect of social support on the relationship between stress and depression exists among medical staff [41]. Furthermore, social support has a moderating effect on the relationship between burnout and mental health stigma [25]. However, for the first time, the mechanism underlying the association between social support, burnout, and anxiety symptoms in this population was analyzed in this study. The results show that social support can moderate the stress process due to the buffering effects. Social support may play a vital role at two different points in the process linking burnout to anxiety symptoms [18]. First, individuals experiencing burnout may have the perception that others will offer important resources, so this can help individuals redefine the potential for harm posed by burnout. Resources from social networks can enable individuals to have a sense of predictability and stability in their lives, as well as a recognition of self-worth, personal control, and mastery. Therefore, they are less likely to be affected by burnout [18]. Second, social support has the potential to reduce the likelihood of developing anxiety symptoms in the stress process, since support resources can mitigate the impact of burnout by providing problem solving strategies such as material support and intangible resources [18]. These strategies can reduce the intensity of the relationship between burnout and anxiety. Although the moderating effect of social support on the relationship between burnout and anxiety symptoms was statistically significant, the effect size was quite small limiting practical significance of data. Therefore, the buffering capabilities of social support should not be overestimated.

In this study, 56.03% of participants experienced burnout, which was higher than the findings from a Singapore study (11%) [42]. Additionally, this study suggested that 48.25% of participants experienced anxiety symptoms. In contrast, another Chinese study showed that the prevalence of anxiety symptoms among ICU medical staff was 35.2% [43]. Our study found that the prevalence of anxiety symptoms and burnout are common in ICU. One possible explanation may be that critically ill patients require medical staff to effectively assess, monitor, and respond to their needs by adopting specialized knowledge and advanced skills [44]. Another reason may be that although Chinese ICU medical staff must deal with the demanding workload, they always receive criticism and dissatisfaction about their professional performance. These negative attitudes often result in workplace violence, which may cause mental health issues among medical staff [45].

## Implications

According to the “Healthy China 2030” program, institute executives should give top priority to screening and prevent burnout and mental health problems among certain professional groups. Therefore, improving mental wellbeing among ICU professionals should be a matter of utmost concern to healthcare policy makers.

The higher ICU staff assess their social support, the lower is the detrimental impact of burnout on anxiety symptoms. Social support can be regarded as an effective and low-cost way to improve mental health among ICU staff with burnout. Therefore, we suggest that a multifaceted social support program based on the Chinese cultural values should be developed to promote mental health among Chinese ICU medical professionals.

In traditional Chinese culture, family is a core value that can be leveraged to promote social support. ICU physicians and nurses should be encouraged to enhance bonds with family members, so these professionals can gain various support resources through seeking intangible resources (e.g., love, reassurance, and safety) and material resources (e.g., financial support). Additionally, ICU departments can increase greater understanding and support between staff and family members through hosting a “visiting day”. In this way, family members can gain first-hand experience of the workplace environment in the ICU, and better understand the duties of the healthcare professionals on the unit.

Another core value in Chinese culture is “harmony” which means mutual aid [46]. Positive interpersonal relationships with colleagues and supervisors are always encouraged in Chinese traditional culture. Therefore, this harmonious relationship should be promoted and fostered in the workplace, as it can be another important source of social support [47]. ICU professionals should offer each other emotional and practical support in challenging circumstances to prevent anxiety symptoms. For example, in the ICU setting, staff can share resources and problem-solving skills with their colleagues who find it difficult to cope with a critical patient due to limited resources. To facilitate this, ward managers or unit leaders can initiate a wide range of team development training to help ICU physicians and nurses create a culture of harmony in the workplace.

## Study limitations

Due to the cross-sectional design of the study, the results should not be interpreted with causal directionality. Although we confirmed the relationships between anxiety symptoms and burnout based on a theory-driven hypothesis and studies show the directionality between anxiety symptoms and burnout [10, 32], we cannot rule out the possibility that anxiety symptoms may predispose an individual for burnout [48]. In some cases, they may play different roles based on different theories. For example, Nauman et al., (2018) found that anxiety was a moderator whereas emotional exhaustion was a mediator [49]. In the future, studies should use a two-wave or three-wave longitudinal design to explore reciprocal relationships between these variables. Second, self-reported questionnaires were adopted in this study, so participant responses may have been influenced by social desirability bias. Third, as the cross-sectional sample was recruited from tertiary hospitals, the findings cannot be generalized in China. Future studies should include other types of hospitals. Lastly, given that there are many potential personal and social resources (e.g., self- efficacy, coping) that could buffer relationships between burnout and anxiety symptoms, we encourage future studies to investigate other moderators in such relationships.

## Conclusion

This study identifies the relationships between social support, burnout and anxiety among ICU professionals and provides a better understanding of the factors that may improve their anxiety symptoms. These findings highlight the importance of various forms of social support to help ICU professionals with burnout avoid suffering anxiety symptoms.

## Data Availability

The datasets used and analyzed during the current study are available from the corresponding author on reasonable request.

## Abbreviations

SAS: Self-Rating Anxiety scale
ULCI: Upper Limit of Confidence Interval
LLCI: Lower Limit of Confidence Interval
AVE: Average variance extracted
DV: Dependent variable
IV: Independent variable
